# Liver Cirrhosis in Pregnancy and Its Fetal Outcomes: A Prospective Case Series

**DOI:** 10.7759/cureus.94189

**Published:** 2025-10-09

**Authors:** Catherine Param Paramamanathan, Krishna Kumar, Kavitha Nagandla, Khai H Wong

**Affiliations:** 1 Obstetrics and Gynecology, Tuanku Ja'afar Hospital, Seremban, MYS; 2 Obstetrics and Gynecology, IMU University, Kuala Lumpur, MYS

**Keywords:** autoimmune hepatitis, chronic hepatitis b, cirrhosis, fetal growth restriction, portal hypertension, pregnancy

## Abstract

Pregnancy in women with cirrhosis is uncommon and carries substantial maternal and fetal risks, yet outcomes may improve with coordinated multidisciplinary care. We prospectively followed three pregnant women with confirmed cirrhosis between January 2023 and June 2024 in a tertiary hepatology-maternal-fetal medicine clinic. Management strategies included disease severity assessment, targeted endoscopy, beta-blockers for portal hypertension, antiviral therapy for hepatitis B, hematologic optimization, and individualized delivery planning. Two women had compensated cirrhosis without portal hypertension, and one had a history of decompensation with persistent portal hypertension. Deliveries comprised two cesarean sections, one for pathological cardiotocography and one for failed trial of labor, and one induced vaginal birth following platelet transfusion. No cases of variceal hemorrhage, hepatic encephalopathy, or maternal death occurred. All neonates were liveborn with Apgar scores of 9 at one and five minutes; one had growth restriction. At six weeks postpartum, all mothers remained clinically stable, and hepatitis B prophylaxis prevented vertical transmission. This case series highlights that multidisciplinary management enables favorable outcomes in selected cirrhotic pregnancies.

## Introduction

Cirrhosis constitutes an advanced stage of chronic liver disease characterized by diffuse fibrosis and architectural distortion that impair hepatic synthetic, metabolic, and hemodynamic functions [1]. The physiologic changes of normal gestation, including plasma volume expansion, increased cardiac output, reductions in systemic vascular resistance, and hormonally mediated adaptations, can intersect with cirrhosis in ways that heighten the risk of portal hypertension exacerbation, coagulopathy, and hepatic decompensation [2]. Fertility impairment in cirrhosis due to hypothalamic-pituitary-gonadal axis dysregulation and hyperestrogenism historically limited the number of pregnancies; however, improved chronic disease control, earlier diagnosis, and broader access to hepatology care have increased the frequency with which compensated patients conceive [3,4]. Consequently, there is renewed clinical urgency to define peri-pregnancy risk, refine surveillance, and detail peri-delivery algorithms that protect both maternal and fetal outcomes.

While the literature suggests overall maternal mortality has declined compared with historical cohorts, cirrhosis in pregnancy remains associated with excess obstetric interventions, higher rates of fetal growth restriction and preterm birth, and an increased probability of hemorrhagic and thrombotic complications owing to rebalanced but fragile hemostasis [5,6]. Data sets integrating granular maternal liver metrics, obstetric process measures, and infant follow-up within a single prospective pathway remain limited [7]. The present prospective case series addresses this gap by reporting harmonized clinical, biochemical, and perinatal variables across three carefully phenotyped cases managed in a tertiary multidisciplinary program. This prospective case series aimed to evaluate maternal and fetal outcomes among pregnant women with cirrhosis managed under a structured multidisciplinary protocol.

## Case presentation

This prospective case series was conducted between January 2023 and June 2024 in a tertiary referral center with a joint hepatology-maternal-fetal medicine clinic using integrated records and standardized care pathways. Consecutive pregnant women with singleton gestation and confirmed cirrhosis, diagnosed through clinical stigmata, laboratory patterns, and imaging, were included. Acute liver failure and multifetal pregnancies were excluded. All three women were referred to our tertiary clinic from obstetric units following abnormal liver function tests or clinical suspicion of chronic liver disease during antenatal care. Each patient presented with compensated status at entry and underwent systematic evaluation upon enrollment.

Baseline assessment comprised complete blood count, liver function tests, international normalized ratio, creatinine, fasting glucose, and HbA1c when indicated, hepatitis B serology with HBV DNA quantification, and autoimmune markers when relevant. Ultrasound with portal venous Doppler assessed hepatic texture, portal diameter/flow, and splenic size. Endoscopy was performed in the second trimester if there was prior variceal bleeding, thrombocytopenia <110 × 10⁹/L suggestive of portal hypertension, or sonographic signs of portal hypertension. Fetal surveillance included serial growth scans every two to four weeks with umbilical and middle cerebral artery Dopplers after 28 weeks if growth restriction was suspected, and cardiotocography from 32 weeks or earlier as needed.

Management strategies included nonselective beta-blockers for established portal hypertension or prior variceal bleeding, tenofovir for hepatitis B with high viral load or HBeAg positivity, and hematology-defined platelet transfusion thresholds before induction or neuraxial anesthesia. Delivery planning generally favored vaginal birth, with efforts to avoid prolonged second stage or difficult instrumentation in portal hypertension; cesarean section was reserved for obstetric or hepatic indications. Postpartum follow-up at two and six weeks included maternal laboratory tests and infant assessments, with hepatitis B-exposed neonates undergoing early serologic monitoring.

Overview of cases

Three women were enrolled during the study period; two had compensated cirrhosis without portal hypertension, while one had compensated cirrhosis with prior decompensation and persistent portal hypertension. Etiologies included idiopathic disease, autoimmune hepatitis, and chronic hepatitis B (Table 1) [8]. All pregnancies were singletons. There were no maternal deaths, episodes of hepatic encephalopathy, intensive care admissions, or antepartum/intrapartum variceal hemorrhage. One pregnancy was complicated by fetal growth restriction, which required delivery for non-reassuring fetal testing. Neonatal outcomes at birth were reassuring (Table 2), and six-week follow-up confirmed maternal biochemical stability and appropriate infant growth (Table 3).

**Table 1:** Baseline maternal characteristics and liver disease severity at enrollment ^*^ Reference ranges per institutional laboratory standards. ^†^ Child-Pugh, MELD, APRI, and ALBI are validated and free-to-use scoring systems [8]. ALBI, albumin-bilirubin; APRI, AST-to-platelet ratio index; MELD, Model for End-Stage Liver Disease

Parameter	Case 1	Case 2	Case 3	Reference range^*^
Age (years)	32	26	36	-
Gravidity/parity	G2P1	G1P0	G6P5	-
BMI (kg/m²)	27	22	38	18.5-24.9
Etiology of cirrhosis	Idiopathic	Autoimmune hepatitis	Chronic hepatitis B	-
Prior decompensation	No	Yes - variceal bleed	No	-
Platelet count (×10⁹/L)	136	58	95	150-400
Child-Pugh score (class)^†^	5 (A)	5 (A)	6 (A)	-
MELD score^†^	9	11	6	-
APRI^†^	0.7	1.1	1.1	<0.7 (low fibrosis risk)
ALBI score^†^	-2.60	-2.10	-2.20	
Portal hypertension	No	Yes	No	-
Endoscopy in pregnancy	Not indicated	Grade 1 varices	Not indicated	-

**Table 2:** Antenatal course, delivery details, and immediate neonatal outcomes ^*^ Reference ranges per institutional laboratory standards. CTG, cardiotocography; FGR, fetal growth restriction; GA, gestational age; HBV, hepatitis B virus; NSBB, nonselective beta-blocker; PPH, postpartum hemorrhage; UA, umbilical artery; VBAC, vaginal birth after cesarean

Domain	Case 1	Case 2	Case 3	Reference range^*^
GA at presentation	28 + 5 weeks	12 + 2 weeks	20 + 0 weeks	-
Principal antenatal issues	Late-onset FGR with abnormal UA Doppler	Thrombocytopenia, portal hypertension	Obesity, hyperglycemia, high HBV DNA	-
Key medical therapy	Surveillance only	Propranolol titrated to HR 60-70 bpm	Tenofovir; insulin	-
Hematology peripartum plan	None	Platelet transfusion >80 × 10⁹/L	Avoid neuraxial if platelets <70 × 10⁹/L	Platelets ≥150 × 10⁹/L
Mode of delivery	Emergency cesarean (pathological CTG)	Induced vaginal birth	Cesarean (failed VBAC)	-
GA at birth	36 + 3 weeks	37 + 2 weeks	37 + 3 weeks	≥37 weeks
Estimated blood loss (mL)	650	300	800	<500 vaginal, <1000 cesarean
PPH >1000 mL	No	No	No	-
Neonatal birth weight (g)	1790	2660	3160	≥2500
Apgar 1/5 min	9/9	9/9	9/9	≥7
NICU admission	Yes (five days, feeding support)	No	No	-

**Table 3:** Six-week postpartum maternal and infant outcomes ^*^ Reference ranges per institutional laboratory standards. ALT, alanine aminotransferase; AST, aspartate aminotransferase; HBIG, hepatitis B immunoglobulin; HBsAg, hepatitis B surface antigen; INR, international normalized ratio; NSBB, nonselective beta-blocker

Outcome	Case 1	Case 2	Case 3	Reference range^*^
Maternal clinical status	Asymptomatic, normal exam	Asymptomatic, stable	Asymptomatic, normal exam	-
Platelet count (×10⁹/L)	138	82	98	150-400
ALT/AST (U/L)	28/26	32/30	27/25	ALT: 0-35; AST: 0-35
Total bilirubin (mg/dL)	0.9	1.1	0.8	0.2-1.2
Albumin (g/dL)	3.8	3.6	3.9	3.5-5.0
INR	1	1.1	1	0.8-1.2
Antiviral/NSBB status	None	Continuing propranolol	Continuing tenofovir	-
Breastfeeding	Established	Established	Established	-
Infant growth	Appropriate	Appropriate	Appropriate	-
Infant prophylaxis/serology	Not applicable	Not applicable	HBsAg-negative; HBIG + vaccine given	-

## Discussion

This prospective case series, though numerically small, demonstrates that high-fidelity, protocolized care can enable favorable pregnancy outcomes in women with cirrhosis who are predominantly compensated at conception. The key observations from this cohort arise from the intersection of maternal hemodynamics, placental physiology, and peripartum hemostasis, and they translate into practical guidance for clinical teams.

The first central case concerns portal pressure physiology under gestational load. Normal pregnancy increases intravascular volume and cardiac output, which can raise portal inflow [9]. Women with existing portal hypertension are therefore at risk for variceal complications unless portal pressure is tempered [10]. In this series, the woman with historical decompensation and documented portal hypertension remained free of bleeding, a trajectory plausibly attributable to early second-trimester endoscopic assessment, the continuation of nonselective beta-blockade titrated to physiologically appropriate heart rate targets, careful avoidance of factors that acutely increase intra-abdominal pressure during the second stage, and prompt peripartum hemostatic planning. The absence of hemorrhagic events across the cohort should not be seen as a generalizable guarantee of safety. Instead, it underscores the importance of anticipating the portal hemodynamic peak of late pregnancy and creating individualized buffers through medication, endoscopic planning, and labor management strategies.

The second case relates to placental adaptation in the cirrhotic milieu and the manifestation of late-onset fetal growth restriction. Even in compensated disease with near-normal synthetic function, cirrhosis is associated with systemic endothelial dysfunction and altered vasoactive mediator balance [11]. Such endothelial and vasoactive changes may impair placental vascular remodeling and reduce intervillous perfusion [12]. In our first case, elevated umbilical artery resistance with preserved middle cerebral artery flow suggested late placental insufficiency rather than early constitutional smallness [13]. The implication is that compensated status does not immunize against placental disease; therefore, fetoplacental surveillance should be calibrated to hepatic physiology rather than deferred solely on the basis of reassuring maternal laboratory values [14]. A programmatic schedule of serial growth assessment after viability, with Dopplers and readiness to act on non-reassuring fetal testing, is warranted.

The third case emphasizes comorbidity stacking and intrapartum biomechanics. Morbid obesity and poorly controlled diabetes create a biochemical and mechanical context that favors macrosomia, dysfunctional labor, and heightened risk of operative delivery [15]. In the third case, despite controlled portal physiology, labor failed due to arrest of descent, illustrating that the primary determinant of birth route in cirrhotic pregnancies may be non-hepatic when portal hypertension is absent [16]. These comorbidities also amplify thrombotic risk in the postpartum period and may complicate wound healing [17]. The overall trend of maternal stability and favorable neonatal survival observed in our series is comparable to outcomes reported in other prospective cohorts of pregnant women with cirrhosis [5,6].

The anesthetic and hematologic strategies in such patients therefore need to be anticipatory, with proactive plans for venous thromboembolism prophylaxis, glucose management, antibiotic prophylaxis, and, when neuraxial techniques are considered, conservative thresholds supported by real-time platelet counts and clinical bleeding assessment.

Cirrhosis remains linked with higher rates of preterm birth, cesarean delivery, preeclampsia, and growth restriction, a pattern best understood through a unifying pathophysiologic framework. Cirrhosis produces a rebalanced but fragile hemostatic state, chronic low-grade inflammation, and endothelial dysfunction [18]. These factors can impair spiral artery transformation and placental angiogenesis, pushing pregnancies toward maladaptation that clinically materializes as hypertensive disease and growth restriction [19,20]. Simultaneously, clinical teams often select earlier delivery in the presence of thrombocytopenia, rising portal pressures, or non-reassuring fetal status to mitigate catastrophic events, thereby increasing iatrogenic prematurity and cesarean rates [17]. The data here are congruent with that conceptual model: the decision for early delivery in the first case was shaped by non-reassuring fetal testing superimposed on growth restriction, while the cesarean in the third case was driven by mechanical dystocia in the context of metabolic comorbidity.

These observations carry practical consequences for case selection and counseling. Women with Child-Pugh class A status and Model for End-Stage Liver Disease scores under approximately 10 to 12, absent active decompensation, tend to fare well in pregnancy when they adhere to structured protocols [21]. The presence of historical decompensation does not mandate avoidance of pregnancy if the disease is quiescent, but it should trigger intensified surveillance, a low threshold for endoscopy, and a deliberate effort to blunt portal inflow with beta-blockade [22]. For chronic hepatitis B, initiation of tenofovir in women with high viral load or envelope antigen positivity materially reduces the probability of vertical transmission when paired with newborn immunoprophylaxis, and this should be integrated as a standard pillar of care [23,24]. Delivery should remain obstetrically led, with avoidance of prolonged second stage and difficult instrumentation in patients with portal hypertension, yet without reflexive recourse to cesarean when maternal hepatic status is stable and labor is progressing [16,25]. These recommendations are consistent with recent international guidance, particularly the 2023 European Association for the Study of the Liver Clinical Practice Guidelines on the management of liver diseases in pregnancy, which emphasize individualized delivery planning through multidisciplinary coordination [26].

The strengths of this series include prospective data capture, harmonized maternal and neonatal follow-up through six weeks, and the explicit documentation of peripartum hemostatic and anesthetic decision-making that often goes unreported. The primary limitation is the small sample size, which precludes inference beyond descriptive trajectories and limits the detection of uncommon but serious complications. Additionally, elastography and histology were not uniformly available, a reality in many clinical settings but one that can blur staging precision [27]. Despite these limitations, the consistency of maternal stabilization and neonatal vigor, alongside plausible mechanistic explanations for the observed growth and labor patterns, supports the external relevance of the care pathway described.

## Conclusions

Pregnancy can be feasible and may achieve favorable outcomes in appropriately selected women with cirrhosis when a coordinated, protocol-driven pathway is followed. Risk management should begin with preconception or early pregnancy assessment of hepatic reserve and portal physiology, continue through targeted prophylaxis and vigilant fetoplacental surveillance, and culminate in anesthetic and hematologic plans that respect the precarious balance of hemostasis in advanced liver disease. Comorbid metabolic disease and obesity can dominate intrapartum outcomes even when hepatic status is stable, and they should be addressed with equal rigor. Postpartum follow-up is essential both to detect hepatic flares and to confirm the success of neonatal prophylaxis in hepatitis B-exposed dyads. This case series highlights that multidisciplinary management enables favorable outcomes in selected cirrhotic pregnancies.
